# Fetal iGRASP cine CMR assisting in prenatal diagnosis of complicated cardiac malformation with impact on delivery planning

**DOI:** 10.1111/cpf.12566

**Published:** 2019-03-08

**Authors:** Misha Bhat, Kostas Haris, Sebastian Bidhult, Petru Liuba, Anthony H. Aletras, Erik Hedström

**Affiliations:** ^1^ Pediatric Cardiac Center Skåne University Hospital and Lund University Lund Sweden; ^2^ Laboratory of Computing, Medical Informatics and Biomedical ‐ Imaging Technologies School of Medicine Aristotle University of Thessaloniki Thessaloniki Greece; ^3^ Department of Clinical Sciences Lund Clinical Physiology Skåne University Hospital, and Lund University Lund Sweden; ^4^ Department of Biomedical Engineering Faculty of Engineering Lund University Lund Sweden; ^5^ Department of Clinical Sciences Lund Diagnostic Radiology Skåne University Hospital and Lund University Lund Sweden

**Keywords:** cardiac malformation, cardiac MRI, clinical application, fetal cardiology, fetal CMR, iGRASP

## Abstract

Limited visualization of the fetal heart and vessels by fetal ultrasound due to suboptimal fetal position, patient habitus and skeletal calcification may lead to missed diagnosis, overdiagnosis and parental uncertainty. Counselling and delivery planning may in those cases also be tentative. The recent fetal cardiac magnetic resonance (CMR) reconstruction method utilizing tiny golden‐angle iGRASP (iterative Golden‐angle RAdial Sparse Parallel MRI) allows for cine imaging of the fetal heart for use in clinical practice. This case describes an unbalanced common atrioventricular canal where limited ultrasound image quality and visibility of the aortic arch precluded confirming or ruling out presence of a ventricular septal defect. Need of prostaglandins or neonatal intervention was thus uncertain. Cardiovascular magnetic resonance imaging confirmed ultrasound findings and added value by ruling out a significant ventricular septal defect and diagnosing arch hypoplasia. This confirmed the need of patient relocation for delivery at a paediatric cardiothoracic surgery centre and prostaglandins could be initiated before the standard postnatal ultrasound. The applied CMR method can thus improve diagnosis of complicated fetal cardiac malformation and has direct clinical impact.

## Case report

Fetal ultrasound has long been the only modality for *in utero* assessment of anatomy and cardiac function in congenital heart disease (Kleinman *et al*., 1980). Limited acoustic windows due to suboptimal fetal position, patient habitus and skeletal calcification towards term may constrain ultrasound to adequately visualize the heart and large vessels. This may result in inappropriate delivery planning and management and cause parental uncertainty and anxiety (Bratt *et al*., 2015). Advanced cardiac care is increasingly centralized to specialized centres. Confirmed but also uncertain need of postnatal intervention thus necessitates delivery at a paediatric cardiothoracic surgical centre. Relocating families for delivery may also cause stress and inconvenience (Brosig *et al*., 2007). As capacities of these specialized centres are finite careful identification and selection of appropriate patients for referral is crucial. Selection is however challenged by limited cardiovascular visualization by ultrasound or in particular diagnoses with a high rate of false positives (Matsui *et al*., 2008). Self‐gated fetal cine cardiovascular magnetic resonance (CMR) imaging has enabled cine CMR for assessing fetal cardiac anatomy and function also in cases where ultrasound is insufficient for diagnosis (Lloyd *et al*., 2016; Roy *et al*., 2016; Chaptinel *et al*., 2017; Haris *et al*., 2017). One such solution (Haris *et al*., 2017) based on so‐called iterative Golden‐angle RAdial Sparse Parallel (iGRASP) MRI (Feng *et al*., 2014; Wundrak *et al*., 2016) was applied in the presented case showing how this CMR method can improve diagnostic accuracy and have direct impact on delivery planning.

**Figure 1:**
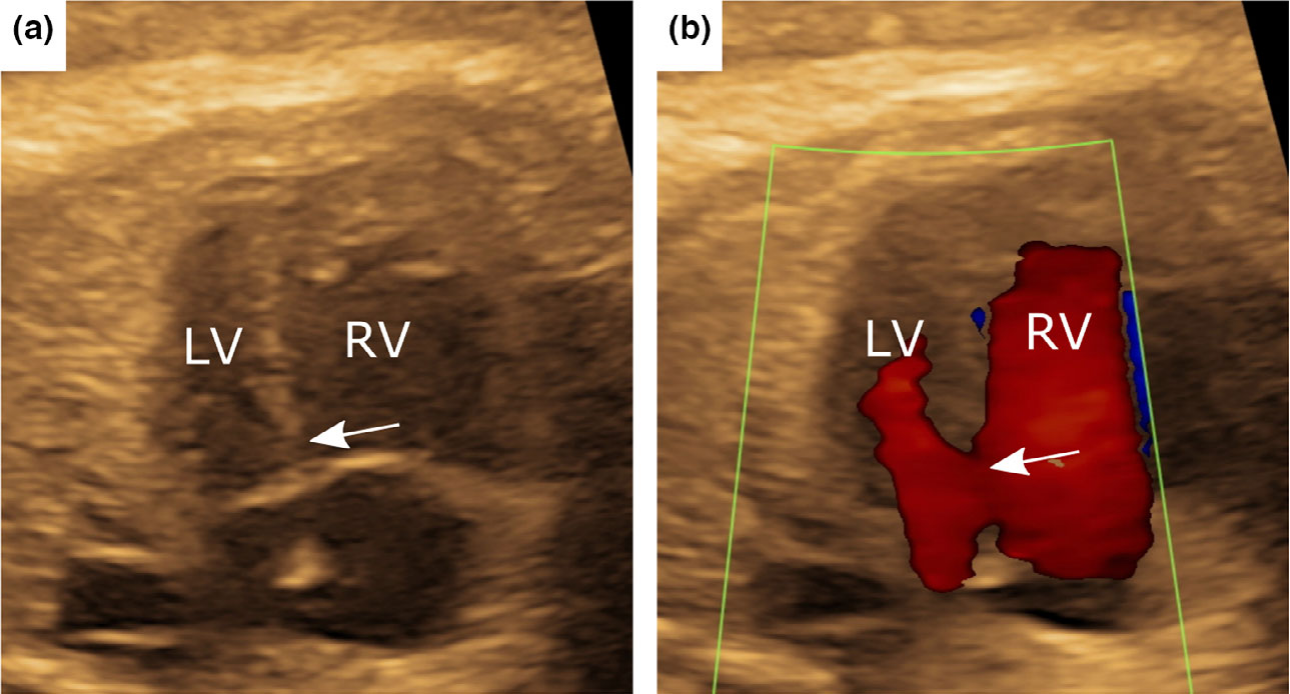
The fetal echocardiogram at 32 + 6 weeks gestational age indicated an unbalanced common atrioventricular canal favouring the right ventricle. Hypoechoic dropout (a) and flow by colour Doppler (b) in the inlet septum region gave the appearance of a possible ventricular septal defect (arrows), but this is a common area for hypoechoic dropout and diagnosis is uncertain. LV = left ventricle; RV = right ventricle.

A 37‐year‐old healthy gravida 2 para 2 pregnant woman was referred from a secondary hospital to the fetal cardiothoracic centre at 32 + 6 weeks gestational age. She was referred due to size discrepancy between the left and right ventricles shown by a late screening ultrasound. The pregnancy was otherwise uncomplicated. Poor acoustic windows due to patient habitus and suboptimal fetal position limited the visualization of the heart and aortic arch. The diagnosis of an atrioventricular canal with unbalance favouring the right ventricle was made. The left ventricle was apex forming, and the aortic valve was normal in size. Presence of a ventricular septal defect was unclear. Although there was both hypoechoic dropout and Doppler signal indicating a potential ventricular septal defect (Fig. 1), the inlet septum is an area of frequent hypoechoic dropout, which creates the appearance of a ventricular septal defect even when there may be none. Further, ventricular size discrepancy is associated with aortic coarctation (Brown *et al*., 1997), important information for managing this case. Ultrasound could however neither confirm nor discard coarctation, as the arch could not be visualized. As the left ventricular outflow tract was well developed, presence or absence of a ventricular septal defect or coarctation would impact postnatal physiology and ductal dependency.

A repeat fetal echocardiogram was performed according to clinical routine at 36 + 0 weeks gestational age. Ultrasound images were of worse quality and presence or absence of a ventricular septal defect, aortic arch hypoplasia or coarctation could not be clarified. The patient was therefore referred for fetal CMR for further assessment of cardiovascular anatomy and function.

Fetal CMR was performed at 37 + 4 weeks gestational age using a 1·5 T Siemens Aera scanner (Siemens Healthineers, Erlangen, Germany). Anatomic balanced steady‐state free‐precession (bSSFP) image stacks (1 × 1 × 4 mm^3^ acquired voxel size) were acquired to assess cardiovascular anatomy and for cine‐view planning. Fetal cardiac cine imaging was acquired using tiny golden‐angle radial sampling combined with iGRASP for accelerated acquisition based on parallel imaging and compressed sensing. This enabled multi‐slice cine‐loop acquisitions of the heart in 4‐chamber and short‐axis views with 1·37 × 1·37 × 4 mm^3^ acquired voxel size (no interpolation) and 29·6 ms temporal resolution. Fetal motion was not an issue, and scans were not repeated.

**Figure 2:**
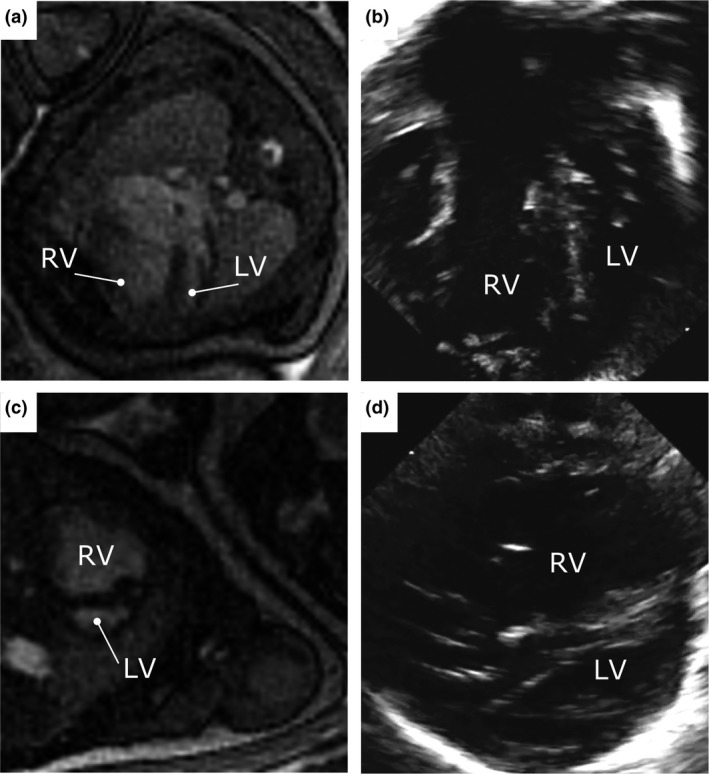
Fetal iGRASP cardiovascular cine magnetic resonance imaging in 4‐chamber (a) and short‐axis (c) views, clearly showing the unbalanced common atrioventricular canal favouring the right ventricle, and ruling out a moderate or large inlet ventricular septal defect or other ventricular septal defect. Images are shown without interpolation. The corresponding postnatal echocardiogram acquired ten days after delivery are shown for comparison in 4‐chamber (b) and short‐axis (d) views, confirming the prenatal diagnosis by iGRASP cardiovascular cine magnetic resonance imaging. LV = left ventricle; RV = right ventricle.

**Figure 3:**
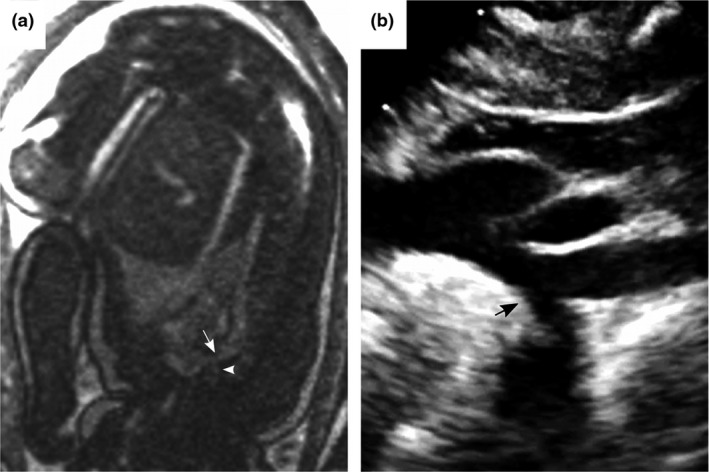
Fetal balanced steady‐state free‐precession (bSSFP) magnetic resonance imaging of the aortic arch (a) showed a hypoplastic aortic arch with minimum diameter 2·8 mm (arrow) in the aortic isthmus. The left subclavian artery is denoted (arrowhead). Images are shown without interpolation. The presence of a hypoplastic arch was confirmed by echocardiography 10 days after delivery (b).

Fetal iGRASP cine CMR imaging confirmed and clearly demonstrated an unbalanced common atrioventricular canal favouring the right ventricle, with preserved ventricular systolic longitudinal and circumferential shortening (Fig 2a and c and Video clips S1 and S2). A moderate/large ventricular septal defect was ruled out by cine CMR. Anatomic image stacks revealed arch hypoplasia with a diameter of 2·8 mm in the aortic isthmus in the sagittal plane (Fig. 3a), corresponding to a *Z*‐score of−2·8 using ultrasound‐based reference values as none have been published for fetal CMR (Pasquini *et al*., 2007). No posterior shelf was seen on CMR but this is not always evident prior to ductal closure and contributes to the challenge of prenatal diagnosis of coarctation and a *Z*‐score of < −2 indicates need of neonatal surgical intervention (Matsui *et al*., 2008).

Altogether, these findings confirmed the need for delivery at a paediatric cardiothoracic centre with the decision to initiate prostaglandins to maintain ductal patency prior to postnatal evaluation by a cardiologist. Furthermore, more definitive information could be given to the parents regarding surgery. The neonate underwent biventricular repair with arch reconstruction at age 10 days, which was successful. A presurgical echocardiogram was performed according to clinical routine and used for comparison with prenatal CMR findings. By accurately showing anatomy and function prenatally, CMR optimized delivery planning to include immediate initiation of prostaglandins rather than awaiting first clinical postnatal assessment. It also confirmed the need for family relocation for delivery at the paediatric cardiothoracic surgical centre. In other cases with limited echocardiographic imaging, better visualization by fetal CMR might even permit delivery at local hospitals. In the current case, presenting a more definitive plan to the parents prenatally allowed for decreased uncertainty and better management of expectations.

The postnatal echocardiogram confirmed the diagnoses proposed by CMR (Figs 2b,d and 3b). Follow‐up at 6 and 12 months of age showed excellent surgical results with no left or right atrioventricular valve stenosis, minimal regurgitation and a patent aortic arch.

## Discussion

This case demonstrates clinical utility of targeted imaging of the fetal heart and vessels using fetal iGRASP cine CMR, and how it may impact delivery planning. Recent technological advancements in fetal CMR allow for accurate diagnosis of cardiac malformation. Whereas ultrasound is widely available and thus a first‐hand choice for screening, CMR can be particularly useful for adding diagnostic value in select patients in whom ultrasound is inconclusive. This carries clinical relevance and impacts delivery planning, allowing for more accurate information to the expecting parents‐to‐be, increased patient safety and improved resource utilization.

Fetal CMR is still limited in spatial and temporal resolution considering the high fetal heart rate, the moving foetus, imaged small structures and distance from coil introducing low signal‐to‐noise ratios. Technical developments are however rapid and recent methods are able to obtain fetal cine CMR images with an acquired inplane spatial resolution of 0·5 mm (Haris *et al*., 2018). Further, as measurements of cardiovascular structures should be performed in end‐diastole or end‐systole improved acquired temporal resolution as provided by direct triggering methods (Kording *et al*., 2018) is important for reproducible and accurate measurements and diagnosis. The lack of a trigger signal has also been one of the major limitations to widely available fetal cine CMR as the standard solution using ECG gating is not possible. With the introduction of an MR‐compatible Doppler ultrasound device providing a signal to the MR scanner for triggered acquisition of fetal CMR data, both acquisition and reconstruction have been simplified as available standard cine CMR sequences can be applied also for fetal cardiovascular diagnosis. Gradient switching, temperature and noise should however be kept to a minimum in fetal CMR, in view of patient safety. Spatial and temporal resolution are thus still limited in relation to fetal heart rate and vessel size. Nevertheless, the current case together with other recent developmental work shows increased availability of fetal CMR for clinical decision‐making. Whereas previous studies have focussed on developmental work, the current case shows clinical utility for prenatal evaluation of cardiovascular malformation with information provided to impact clinical decision‐making.

## Conflict of interest

All authors declare no conflict of interest.

## Ethical approval

All procedures were in accordance with the ethical standards of the Regional Ethical Review Board and with the 1964 Helsinki declaration and its later amendments and approved by the Regional Ethical Review Board.

## Informed consent

Informed consent was obtained from the participant included in the study including for use of images in publication.

## Supporting information


**Video Clip S1.** Fetal iGRASP cardiovascular cine magnetic resonance imaging in a midventricular short‐axis view clearly showing the preserved ventricular systolic circumferential contraction and relatively small left ventricle. The cine loop was acquired using 1·37 × 1·37 × 4 mm^3^ acquired voxel size (no interpolation) and 29·6 ms temporal resolution, within a maternal breath‐hold of 15 s.

**Video Clip S2.** Fetal iGRASP cardiovascular cine magnetic resonance imaging in a 4‐chamber view clearly showing the preserved ventricular systolic longitudinal shortening and common atrioventricular canal defect favouring the right ventricle. The cine loop was acquired using 1·37 × 1·37 × 4 mm^3^ acquired voxel size (no interpolation) and 29·6 ms temporal resolution, within a maternal breath‐hold of 15 s.
